# How to manage celiac disease and gluten-free diet during the COVID-19 era: proposals from a tertiary referral center in a high-incidence scenario

**DOI:** 10.1186/s12876-020-01524-4

**Published:** 2020-11-19

**Authors:** Luca Elli, Donatella Barisani, Valentina Vaira, Maria Teresa Bardella, Matilde Topa, Maurizio Vecchi, Luisa Doneda, Alice Scricciolo, Vincenza Lombardo, Leda Roncoroni

**Affiliations:** 1 Center for Prevention and Diagnosis of Celiac Disease, Gastroenterology and Endoscopy Unit, Fondazione IRCCS Ca’ Granda Ospedale Maggiore Policlinico, Via F. Sforza 35, 20122 Milan, Italy; 2 Department of Pathophisiology and Transplantation, University of Milano, Milan, Italy; 3 School of Medicine and Surgery, University of Milano-Bicocca, Monza, Italy; 4 Division of Pathology, Fondazione IRCCS Ca’ Granda Ospedale Maggiore Policlinico, Milan, Italy; 5 Department of Biomedical, Surgical and Dental Sciences, University of Milan, Milan, Italy

**Keywords:** Celiac disease, COVID-19, SARS-CoV-2, Gluten-free diet

## Abstract

The outbreak of COVID-19 and SARS-CoV-2 infection is spreading worldwide as the first coronavirus pandemic. The clinical picture is variable but flu-like symptoms are common with bilateral interstitial pneumonia being the most frightening presentation. No specific therapies nor vaccine have been developed to date and the only way to limit the virus diffusion is by modifying one’s lifestyle limiting social life and following strict hygienic precautions. No data is available on the risk of COVID-19 and its outcomes in celiac disease (CeD). The restrictions applied to counter COVID-19 can impact on CeD treatment and gluten-free dieting, the only available therapy for CeD. With the present manuscript, we aim to support gastroenterologists and nutritionists in the management of CeD patients in the new pandemic scenario, being conscious that availability and local situations are extremely various.

## Background

During the dramatic coronavirus (COVID-19) pandemic, many Italian patients affected by celiac disease (CeD) have asked gastroenterologists about their possible risk of SARS-CoV-2 infection and if their condition would in any way make the lung disease worse [1]. Actually, little is known about Severe Acute Respiratory Syndrome—CoronaVirus 2 (SARS-CoV-2) infection, especially among Western populations, and if and how subjects with an on-going autoimmune disorder (e.g. CeD) [2] are affected. From this point of view, CeD is considered a long-life autoimmune disorder of the small bowel (SB) caused by the ingestion of gluten-containing food in genetically susceptible subjects carrying the HLA DQ2 and/or DQ8 haplotypes and affecting approximately 1% of the general population [3]. The main hallmarks of CeD are the serological presence of autoantibodies (anti tissue transglutaminase and anti endomysial IgA) and duodenal atrophy, characterised by villous shortening, crypt hyperplasia and increased intra-epithelial lymphocytes (IELs) [4]. Being one of the most common autoimmune diseases (approximately 0.5% of the general population can be affected by CeD) [5], the possibility of any interaction between SARS-CoV-2 infection and the immune system of CeD subjects could be clinically and epidemiologically relevant. Nowadays, no data and no indications are present about this important issue.

The present study aims to give practical indications derived from the real life of a single CeD center (the Center for Prevention and Diagnosis of Celiac Disease, Fondazione IRCCS Ca’ Granda, Milan) in a COVID-19 high-incidence area and convey some proposals to stratify CeD patients.

## SARS-CoV-2 infection and interactions with the gluten susceptible genetic and inflammatory environment

SARS-CoV-2 was identified as the causative pathogen of an interstitial pneumonia outbreak in Wuhan (China). SARS-CoV-2 is characterized by a non-segmented, single-strand, positive-sense RNA genome; human-to-human transmission fuelled by respiratory droplets represents the main cause of inter-human diffusion, although a fecal–oral route and transmission by fomites cannot be ruled out [6–8]. Angiotensin-converting enzyme 2 receptor (ACE2) protein is the cell receptor for SARS-CoV-2 entrance into host cells [7]. The COVID-19 clinical picture being dominated by flu-like symptoms, and illness is more severe in cases of older age, smoking and presence of co-morbidities (e.g. chronic obstructive pulmonary disease, diabetes, hypertension, coronary heart disease, cerebrovascular disease, hepatitis B infection, cancer, chronic renal disease, immune-deficiency). To diagnose COVID-19, the reverse transcription polymerase chain reaction (RT-PCR) of specimens from the respiratory tract is recommended [9].

Different viral and celiac biological factors could potentially interact. Starting from genetics, the combination of HLA-DQA1*0501 and DQB1*0201 generates the HLA-DQ2.5 heterodimer, present in more than 90% of CeD patients, whereas the remaining patients carry the HLA-DQ8 heterodimer, encoded by DQA1*03 (α chain) and DQB1*0302 (β chain) [10]. HLA class II molecules are essential for antigen presentation to CD4 + T helper lymphocytes, since the combination of the α and β chains creates a groove that hosts the peptides, which are going to trigger the T-cell response [11]. Although HLA-DQ2 has a reduced ability to interact with co-factor DM compared to other class-II molecules [12], suggesting a deteriorated presentation of the antigen to T helper cells and this, in turn, could influence the antiviral response of DQ2 positive individuals, up to now there is very contrasting data on the presence of an altered immune response to viruses in CeD patients. Moreover, it should be remembered that viruses are preferentially presented through HLA class I molecules and that the DQ2 heterodimer is present in about 25%-30% of the Caucasian population, and thus possible alterations due to the presence of this specific HLA could affect not only CeD patients but also other carriers [11]. Although CeD patients show an immune response comparable to that of the general population, the hepatitis B virus (HBV) vaccine is the only exception to this statement [13]. The reason of HBV vaccination’s failure in CD patients has not yet been thoroughly explained but polymorphisms in the human leukocyte antigen (HLA) have a crucial role [13].

A very recent and interesting *in-vitro* study has analyzed the role of DM in the processing of various peptides bound to DQ2, and the resulting activation of specific T-cell clones; apart from gluten peptides, the study has evaluated viral peptides derived from HSV type 2, hemagglutinin protein from H1N1 and human papillomavirus type 16 (HPV16). In presence of high expression of DM in DQ2 antigen-presenting cells, the activation of the T-cell response to the virus peptides was increased, and this could be important since some virus infections are able to “naturally” raise DM expression in the infected cells [14]. The presence of a specific HLA associated with a more severe COVID-19 infection has also been investigated in the attempt to identify possible predictive factors. However, the results have not clarified the issue, since different inclusion criteria and endpoints have been evaluated. A small study identified an association between some specific HLA (B*27:07, DRB1*15:01 and DQB1*06:02) and the presence of COVID-19 infection [15], whereas a larger study, which used a GWAS approach to identify severity-associated genes and performed on Italian and Spanish patients, did not report a significant role of the HLA region (Ellinghouse et al.) [16]. Interestingly, in the same study, two regions were detected as significantly associated with a more severe clinical picture, namely the AB0 blood group, and the 3p21.31 locus which harbors a cytokine/chemochine cluster. Interestingly, a locus predisposing to CeD (CELIAC9) has been mapped to 3p21, but the COVID-19 risk allele GA of the polymorphism rs11385942 is associated with reduced expression of CXCR6 and increased expression of SLC6A20, such genes being different from those directly involved in CeD.

CeD is generally characterized by a pro-inflammatory status and variants within the CTLA4, IL2, IL18 and IL21 loci have been associated with CeD susceptibility [11, 17]. CeD-associated inflammation is characterized by high IL21, IFNγ and IL17a [18]. Up-regulation of IL15 expression in the SB mucosa has become a hallmark of active CeD. Moreover, IL15 stimulates IL21 production in the enteric mucosa, further sustaining a pro-inflammatory environment. A question is raised here: in the COVID-19 pandemic may CeD-associated inflammation contribute to a higher degree of susceptibility to SARS-CoV-2 infection or predispose to more severe viral infection? Recent evidence suggests that a subgroup of patients with severe COVID-19 infection shows cytokine storm syndrome [19]. COVID-19 infection is characterized by increased IL2, IL7, granulocyte-colony stimulating factor, IFNγ inducible protein 10, monocyte chemoattractant protein 1, macrophage inflammatory protein 1α, and TNFα. Moreover, higher levels of IL6 were shown to be predictive of COVID-19 death, further suggesting that mortality was associated with hyper-inflammation and cytokine storm [20]. Whether the perturbation of ILs prompted by the viral infection worsens symptoms in CeD patients, or interacts with the pre-existent inflammatory status, is unknown. Further molecular analysis is needed to clarify if the variants in CeD-associated inflammatory factors are associated to any increased risk of a more severe COVID-19 status.

Factors potentially influencing SARS-CoV-2 infection in CeD patients is reported in Table 1.

**Table 1:** Factors that theoretically could influence SARS-CoV-2 infection in celiac disease

HLA status	There is no data suggesting that there is an altered immune response against SARS-CoV-2 virus
Immunological environment and hyposplenism	There is no evidence that ILs status or their genetic variants in CeD could have any influence. Similarly, hyposplenism may not be considered as a risk factor in this case
Mucosal atrophy	In case of treated and responsive CeD the mucosal state does not seem to have a role
Malabsorption and/or micronutrients deficiencies	Vitamins deficit may lead to increased susceptibility to infections. Although there is no evidence concerning COVID-19, verify the nutritional state and ensure that their alimentary intake is reasonable
Presence of a refractory celiac disease	The presence of this state may significantly worsen the COVID-19 outcome, inflammatory damage and malabsorption being usually present in a severe form

## Factors promoting infection susceptibility in celiac patients

It is critically uncertain whether in 2020 CeD subjects present a higher generic risk of infection as compared to the general population and, if present, it is extremely low. This is due to the absence of novel studies investigating recently diagnosed populations; most of the published data is about patients who lived and were diagnosed some decades ago when diagnostic criteria and patient phenotypes were so far different from the current ones. Moreover, the published databases are not able to exclude patients affected by refractory CeD (RCeD), a subset of patients carrying an extremely high mortality risk due to malignancy and infective events. However, different studies have investigated this issue with conflicting results. Overall, the slightly increased mortality of CeD (OR 1.24, 95% CI 1.19–1.30) is principally fuelled by the increased risk of lymphoma (mainly intestinal lymphoma) [21]. The literature has focused its attention on the risk of bacterial infection; a general increased risk of sepsis has been found in CeD (HR 1.6, 95% CI 1.20–1.90) apparently linked to pneumococcal infection (HR 2.5, 95% CI 1.20–5.10) [22]. Furthermore, an increased risk of tuberculosis in CeD has been occasionally reported (HR 3.74, 95% CI 2.14–6.53) [23]. It is actually extremely uncertain whether these findings lead to increased mortality. The data about viral infection appears more controversial with some recent studies indicating the absence of risk to develop influenza or herpes zoster, independently of the presence of villous atrophy as a bio-marker of active disease [24].

The physio-pathological mechanism underlying this theoretical infection risk in CeD seems mainly connected to hyposplenism. The immune role played by the spleen is extremely important mostly in maintaining the pool of IgM memory B-cells (CD27+) which develop in the spleen marginal zone. Memory B-cells warrant a T-independent response against encapsulated bacteria through IgM antibodies production, mainly *Streptococcus pneumoniae*, *Neisseria meningitidis* and *Hemophylus influenzae* [25]. The estimated prevalence of hyposplenism in CeD patients is 19–80%, with some studies reporting an association between hyposplenism and other autoimmune diseases and malignant complications in CeD patients [26]. Hyposplenism affects only adult CeD patients and it seems to correlate with gluten exposure. In fact, the longer the patient has been exposed to diets containing gluten, the higher is the probability of hyposplenism [25]. For the aforementioned reasons the clinical course of CeD patients with hyposplenism is often characterized by complications and more severe progression. Hyposplenism may be defined by low IgM memory B-cells as well as increased pitted red cells (> 4%) and Howell-Jolly bodies, which are consequences of a defective spleen filtering function of erythrocytes [27]. However, given the difficulty to test such parameters, abdominal ultrasound is a valid substitute, because of the existing association between a small spleen (< 7–8 cm) and reduced splenic function [28]. Nevertheless, today there are no guidelines supporting physicians to evaluate the splenic function systematically in CeD patients, due to data inconclusiveness. Furthermore, the international CeD guidelines do not include pneumococcal vaccination. An interesting exception is represented by the British Society of Gastroenterology, which in 2014 introduced such vaccination as a grade-C recommendation for CeD patients [29].

A defective nutritional status is frequently observed in CeD patients [30]. Folate, vitamin B_12_ and vitamin D play the most important role among all nutrients in affecting the immune system. In fact, the lack of them is associated with a higher risk of respiratory infections (e.g. influenza) and a generally reduced immunocompetence [31]. It is doubtful if mild iron-deficiency anemia (frequently present in CeD) would be a risk factor for viral infections. Moreover, CeD patients generally show an increased intestinal permeability, which has been held responsible for increasing the risk of multiple organ failure in critically ill patients [32]. Such a connection has not yet been described for CeD patients, but it is conceivable that such a condition helps the pathogens’ entry. Emilsson et al. [24] have showed that the healing of intestinal mucosa or persistent villous atrophy after GFD do not seem to influence the risk of infection. The increased permeability seems to affect not only the intestinal mucosa but also the respiratory tract, which may as well increase the risk of airborne infections [32].

A special scenario is represented by RCeD, a rare CeD complication characterized by malabsorption and villous atrophy despite a strict GFD [29]. RCeD can be subdivided in two forms (RCeD-1 and RCeD-2) on the basis of the immunophenotype of intra-epithelial lymphocytes (IELs), RCeD-2 being the more severe. RCeD symptoms are diarrhea, involuntary weight loss, general malaise, anemia, hypoalbuminemia, vitamin deficiencies and concomitant autoimmune disorders [33]. Malnutrition due to protein-losing enteropathy can be relevant, compromising the patient’s performance status. Furthermore, RCeD patients are usually treated with immunosuppressive drugs [29]. These aspects make RCeD at risk of infective complications the same way as other conditions requiring chronic immune suppression, such as inflammatory bowel disease (IBD) or transplanted patients [34–36]. Although not conclusive, the data about COVID-19 and RCeD do not report any increased risk in those fragile patients [37]. This issue could be addressed by the use of an international epidemiological registry, actually absent.

**Fig. 1:**
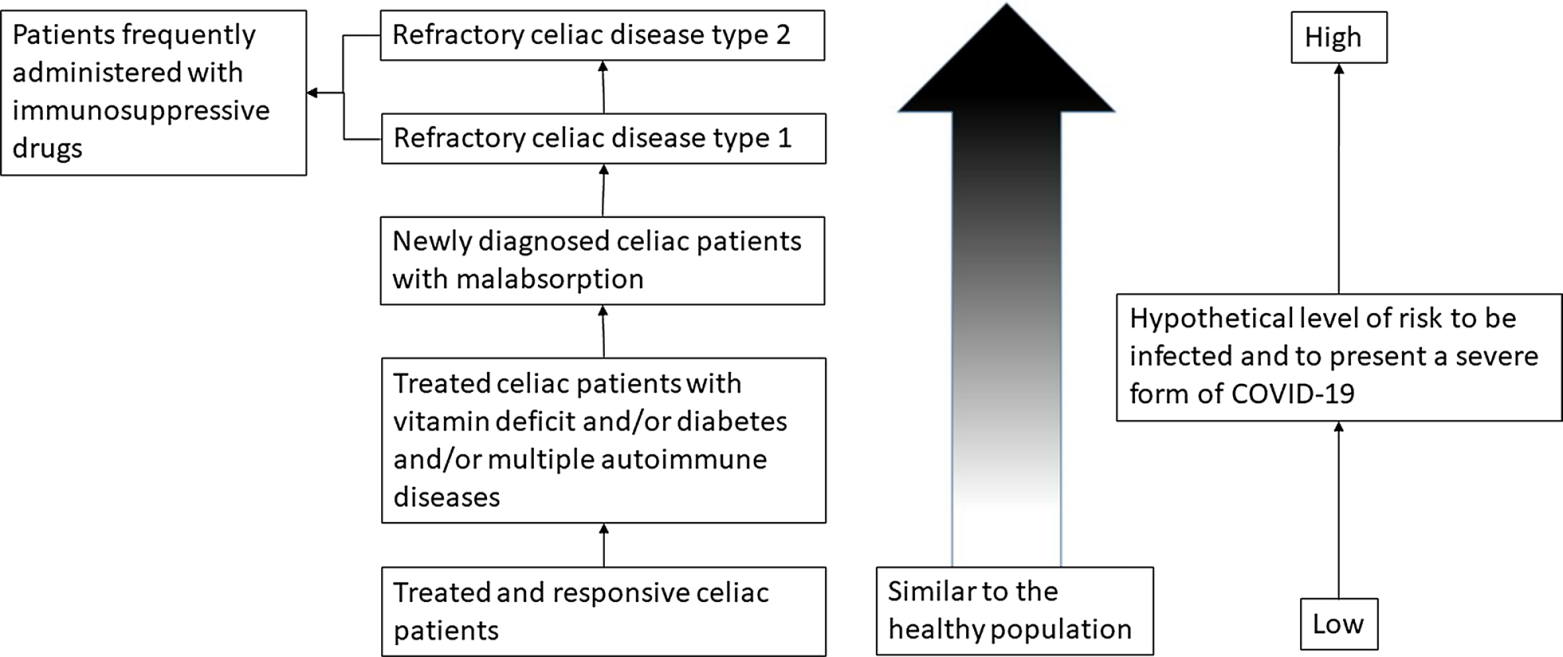
Risk assessment for SARS-CoV-2 infection and development of COVID-19 in celiac patients

Albeit difficult to draw, a theoretical risk stratification of CeD patients for SARS-CoV-2 infection has been reported in Fig. 1.

## Lifestyle and gluten-free dietary advice

In the present COVID-19 crisis the main solution adopted to contain viral spread has been social distancing or quarantine. Staying at home is a good way to have time to “eat well and stay well”, and follow a correct gluten-free Mediterranean diet (MeD-GFD) [38]. In Italy the dietary pattern is characterized by MeD traditions, which provide for the daily consumption of a large amount of wholegrains, plant foods (vegetables, fresh fruits, legumes, nuts, olive oil, herbs and spices), fish, a moderate intake of dairy products, meat, eggs, sugars and wine [39]. MeD has contributed to protect and prevent against such chronic diseases as cardiovascular, respiratory diseases, type-2 diabetes, cancer and obesity, and has been associated with scarcer all-cause mortality rates [40, 41]. Some of the positive effects in which the MeD is involved, regard the intake of phytochemical compounds present in vegetable foods, such as antioxidants [42, 43]. They are metabolized by the intestinal microbiota and are thought to modulate the gut barrier function and immune response, helping to build strength against pathogenic microbes [44–46]. Some of the important molecules for their antioxidant action are: glutathione, ascorbic acid, vitamins A, D, E, polyphenols, omega-3, zinc, selenium, amino acids [47]. As well as vitamins, polyphenols, omega-3, a few minerals (zinc, selenium) and amino acids (glutamine, arginine, tryptophan) found in foods, have immunostimulant activity of potential help [48, 49]. In this context it appears essential to follow a balanced, healthy, MeD, because strong imbalances in the diet, as well as treatment with antibiotics or infections, can disturb the balance of the intestinal microbiota, so as to alter its composition and promote the growth of potentially pathogenic constituents. This condition, known as dysbiosis, can also cause alterations in the regulation of the immune system, which normally prevents the development of inflammatory reactions in our gut; dysbiosis is very often associated with inflammation [50]. As a consequence, it is essential to monitor MeD-GFD [38]; the advice provided by nutritionists is always to prefer a “natural” GFD, avoiding the processed GF foods, especially in the current COVID-19 scenario. It is recommended to choose the entire variety of GF cereals and pseudo-cereals: rice, maize, buckwheat, quinoa, amaranth, which can be consumed as wholegrains and/or can be used to make flour to cook home-made gluten free bread, cake, biscuits, pizza. These products should be preferred and chosen to limit the ultra-processed/industrial GF foods, during this lockdown period for which home stay for social distancing, limited supermarket visits and keeping a balanced healthy diet and domestic lifestyle are recommended. To keep healthy in the context of a GFD, it is correct to include: milk and dairy products, preferring partially skimmed milk, yoghurt and light cheeses, in particular yoghurt is a great source of natural probiotics together with such cheeses as mozzarella, robiola, ricotta, cottage cheese (‘crescenza’, ‘fior di latte’), goat cheese, feta; white meat (chicken, rabbit, turkey) and less frequently red meat (beef and pork); fish, preferring codfish, seabass, sardines, anchovies, limiting swordfish and tuna and cephalopods, squid and octopus. As to legumes, it is advisable to try and increase their consumption and to associate them with cereals as a main course; to have a high fibre intake through fresh fruits and vegetables, up to by a total of 5 portions per day; to use extra-virgin olive oil (EVO) and limit salt, promoting instead the use of herbs and spices. All the listed naturally GF foods are easily available in supermarkets, an important service during forced- isolation conditions (with the advice to limit one’s food shopping frequency to once a week).

Performing physical exercise every day is crucial for a correct lifestyle. In order to contain the virus in this global pandemic it is important to do exercise at home, to maintain a physical routine and to limit any damage due to sedentary behaviour, such as spending long time sitting (smart working, watching television, playing videogames, using mobile devices). Exercise can be various: walking around the house, stairs or ladder climbing, weight-lifting, squats, strengthening, stretching, balance and control, yoga or use of aerobic equipment such as a treadmill or cyclette. Spending this critical period in a safe home environment may also contemplate some practice of e-health, such as video-exercises from the TV or via mobile devices as good help for fitness and mental health. It may suffice to spend 30 min on moderate physical activity or 20 min on intense physical exercise, everyday [51].

## Monitoring gluten-free dieting and clinical status during SARS-CoV-2 infection

A very useful method to support and monitor CeD patients in quarantine or during lockdown may be through telemedicine, which is a set of medical and computer-based techniques to deliver healthcare to patients remotely [52, 53]. As demonstrated by Siniscalchi et al*.* [54] this type of consultation appear to be positively accepted by CeD patients during COVID-19 outbreak. In spite of the reduction of face-to-face consultations as required for containing the risk of infection spreading and because healthcare facilities can be a means of contagion and nutritional services must be maintained during quarantine, “tele-nutrition” appears to be a useful tool. There has been interest in video consultations, which are already being tested in many countries as part of their national digital health strategies [55]. In the literature it is evident that the video-consulting of patients at home as regards nutritional counselling may be appropriate, because personal examination is not strictly necessary and nutritional advice can be easily offered to patients during the video face-to-face consultation. Nowadays, for nutritional counselling, as well as for many other domains, video-consultation may offer a strong opportunity to handle patients in the mid of a great crisis. Furthermore, the availability of novel point-of-care (POC) testing may be of help during telemedicine counselling. Patients can verify the correctness of their GFD by themselves via POC tests that detect the presence of gluten peptides in urine [56], They can then discuss the test result with their nutritionist and/or gastroenterologist during a later telemedicine session. These tests can also be repeated during lockdown and quarantine to verify GF products of uncertain quality. CeD activity can be monitored by means of POC tests detecting anti transglutaminase and/or anti gliadin antibodies, routinely considered as non-invasive bio-markers to monitor the CeD activity status [57, 58]. In case of suspected CeD complications and impossibility to hospitalise the patient, endoscopic investigation of the small bowel (where usually CeD complications are localised) may be carried out via a panoramic-view endoscopic capsule with no external recording system (Capsocam System, USA) [59, 60]. Such a capsule can be sent to the patient, ingested and resent to the gastroenterologist after expulsion in the stools, without need to travel or invasive investigations causing the emission of potentially infective droplets [60]. Furthermore, thanks to its sensitivity in detecting small-bowel mucosal atrophy, capsule endoscopy may also be used to diagnose CeD without upper endoscopy and duodenal biopsy [59, 61]. In case of anti tissue transglutaminase IgA 10x, a no biopsy/endoscopy strategy to diagnose CeD in adults could be used (similarly to the pediatric guidelines) [62, 63]; this possibility has been recently suggested by the British Society of Gastroenterology BSG to reduce the number of upper endoscopies during COVID-19 outbreak (https://www.bsg.org.uk/covid-19-advice/covid-19-specific-non-biopsy-protocol-guidance-for-those-with-suspected-coeliac-disease/).

**Fig. 2:**
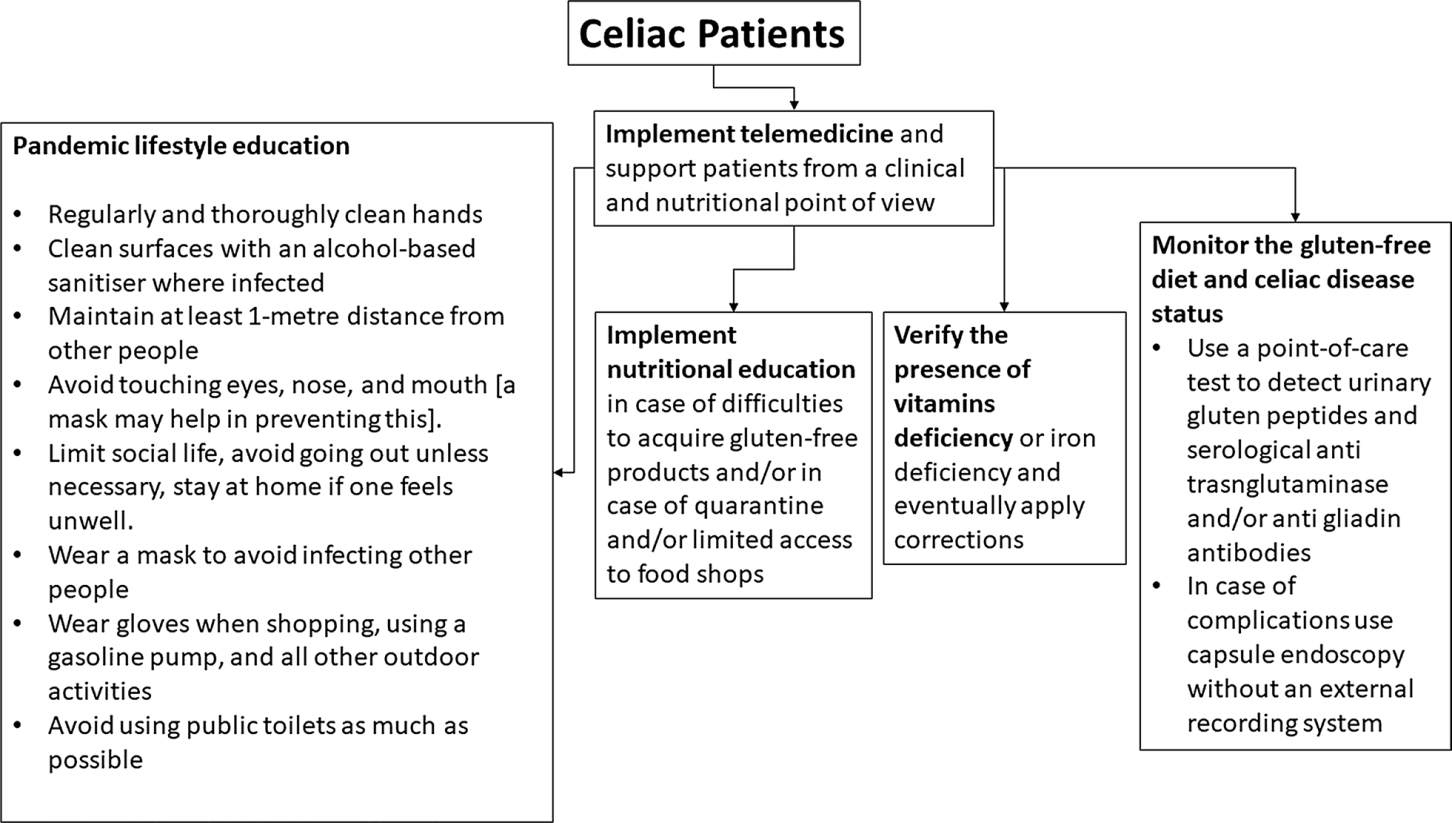
Flowchart for managing CeD patients during the pandemic

A roadmap to manage CeD patients at COVID-19 times is proposed in Fig. 2.

## Conclusions

With the present manuscript we intend to help gastroenterologists and nutritionists in supporting their CeD patients during the COVID-19 outbreak. In Table 2 some key questions and possible answers are provided. We are conscious about the peculiarity of the historical period and that indications and organizational arrangements may change according to the local availability of resources and the specific national health system. However, it is our opinion that the general indications derived from a CeD center managing more than 3,000 CeD patients in an area heavily hit by SARS-CoV-2 infection may be of assistance to those practitioners who unfortunately are facing the same troublesome situation. The first step regards the stratification of patients, distinguishing those who can be managed by means of telemedicine (online systems can be different and depending on local resources) and be educated about lifestyle and GFD dieting during social distancing or over possible quarantine (Figs. 1 and 2). From this point of view, a rapid online service to address the patients’ doubts about GFD should be implemented together with the use of POC tests for urinary gluten peptides and serological antibodies. If feasible, face-to-face online sessions should be devoted to those patients starting GFD at this emergency time. Based on evidence the administration of vitamins to patients presenting a multiple-vitamin deficit is important. Particular attention should be paid to those patients who are affected by RCeD-1 or RCeD-2, following an immunosuppressive therapy: such patients should be closely monitored by means of telemedicine and hospital access should be limited as much as possible, with blood tests and endoscopic investigation postponed whenever possible or being carried out by capsule endoscopy.

**Table 2:** Key-question and answers

Question	What do I tell to the patients
General COVID Advice	Actually there is no evidence that celiac disease represents a COVID-19 risk factor. Proven risk factors for COVID-19 remain old age, hypertension, diabetes, coronary artery disease, pulmonary disease, chronic kidney disease, high body mass index
What about hyposplenism	Reassure patients that functional hyposplenism does not pose any greater risk
Refractory celiac disease	Although unknown, patients with refractory celiac disease and/or taking immunosoppressive/chemotherapic agents may present an increased risk for COVID-19; thus, they should pay attention to distancing and shielding
Telecon clinics	In all cases telemedicine and gastroenterological/nutritional video-consulting can support the patient
Dietary advice including Mediterranean and gluten-free dietary regimens	Improve the diet, in particular follow a gluten-free Mediterranean diet, increasing the intake of antioxidant micro-nutrients

## Data Availability

Not applicable.

## Abbreviations

CD: Celiac disease
COVID-19: Coronavirus disease 2019
GFD: Gluten-free diet
RCeD: Refractory celiac disease
SARS-CoV-2: Severe acute respiratory syndrome (from) coronavirus 2

## References

[1] Sun K, Chen J, Viboud C. Early epidemiological analysis of the coronavirus disease (2019). outbreak based on crowdsourced data: a population-level observational study. Lancet Digit Health.

[2] Lu R, Zhao X, Li J, Niu P, Yang B, Wu H (2020). Genomic characterisation and epidemiology of 2019 novel coronavirus: implications for virus origins and receptor binding. Lancet.

[3] Elli L, Ferretti F, Orlando S, Vecchi M, Monguzzi E, Roncoroni L (2019). Management of celiac disease in daily clinical practice. Eur J Intern Med.

[4] Elli L, Zini E, Tomba C, Bardella MT, Bosari S, Conte D (2015). Histological evaluation of duodenal biopsies from coeliac patients: the need for different grading criteria during follow-up. BMC Gastroenterol..

[5] Ludvigsson JF, Murray JA (2019). Epidemiology of celiac disease. Gastroenterol Clin N Am.

[6] Yang Y, Peng F, Wang R, Guan K, Jiang T, Xu G (2003). The deadly coronaviruses: the 2003 SARS pandemic and the 2020 novel coronavirus epidemic in China. J Autoimmun..

[7] Xiao F, Tang M, Zheng X, Liu Y, Li X, Shan H. Evidence for gastrointestinal infection of SARS-CoV-2. Gastroenterology. 2020;E-pub ahead. 10.1053/j.gastro.2020.02.055 PMC7130181 32142773

[8] Gu J, Han B, Wang J. COVID-19: gastrointestinal manifestations and potential fecal-oral transmission. Gastroenterology. 2020;E-pub ahead. 10.1053/j.gastro.2020.02.054 PMC7130192 32142785

[9] Cascella M, Rajnik M, Cuomo A, Dulebohn SC, Di Napoli R. Features, evaluation and treatment coronavirus (COVID-19). StatPearls Publishing; 2020. https://www.ncbi.nlm.nih.gov/pubmed/32150360. Accessed 20 Mar 2020. 32150360

[10] Sollid LM, Thorsby E (1993). HLA susceptibility genes in celiac disease: genetic mapping and role in pathogenesis. Gastroenterology.

[11] Schuppan D, Junker Y, Barisani D (2009). Celiac disease: from pathogenesis to novel therapies. Gastroenterology.

[12] Nanda NK, Sant AJ. DM Determines the cryptic and immunodominant fate of T cell epitopes. 2000. https://www.jem.org/cgi/content/full/192/6/781. 10.1084/jem.192.6.781 PMC2193287 10993909

[13] Anania C, Olivero F, Spagnolo A, Chiesa C, Pacifico L (2017). Immune response to vaccines in children with celiac disease. World J Gastroenterol.

[14] Hung S-C, Hou T, Jiang W, Wang N, Qiao S-W, Chow I-T (2019). Epitope selection for HLA-DQ2 presentation: implications for celiac disease and viral defense. J Immunol.

[15] Novelli A, Andreani M, Biancolella M, Liberatoscioli L, Passarelli C, Colona VL (2020). HLA allele frequencies and susceptibility to COVID-19 in a group of 99 Italian patients. HLA.

[16] Ellinghaus D, Degenhardt F, Bujanda L, Buti M, Albillos A, Invernizzi P (2020). Genomewide Association Study of Severe Covid-19 with respiratory failure. N Engl J Med.

[17] Van Heel DA, Franke L, Hunt KA, Gwilliam R, Zhernakova A, Inouye M (2007). A genome-wide association study for celiac disease identifies risk variants in the region harboring IL2 and IL21. Nat Genet.

[18] Sarra M, Cupi ML, Monteleone I, Franzè E, Ronchetti G, Di Sabatino A (2013). IL-15 positively regulates IL-21 production in celiac disease mucosa. Mucosal Immunol.

[19] Mehta P, McAuley DF, Brown M, Sanchez E, Tattersall RS, Manson JJ (2020). COVID-19: consider cytokine storm syndromes and immunosuppression. Lancet.

[20] Ruan Q, Yang K, Wang W, Jiang L, Song J (2020). Clinical predictors of mortality due to COVID-19 based on an analysis of data of 150 patients from Wuhan, China. Intensive Care Med..

[21] Tio M, Cox MR, Eslick GD (2012). Meta-analysis: coeliac disease and the risk of all-cause mortality, any malignancy and lymphoid malignancy. Aliment Pharmacol Ther.

[22] Ludvigsson JF, Olén O, Bell M, Ekbom A, Montgomery SM (2008). Coeliac disease and risk of sepsis. Gut.

[23] Ludvigsson JF, Wahlstrom J, Grunewald J, Ekbom A, Montgomery SM (2007). Coeliac disease and risk of tuberculosis: a population based cohort study. Thorax.

[24] Emilsson L, Lebwohl B, Green PHR, Murray JA, Mårild K, Ludvigsson JF (2018). Mucosal healing and the risk of serious infections in patients with celiac disease. United Eur Gastroenterol J.

[25] Casella G, Ingravalle F, Abbate G, Monti C, Bonetti F, Bassotti G (2019). Pneumococcal vaccination in celiac disease. Expert Rev Gastroenterol Hepatol.

[26] Di Sabatino A, Rosado MM, Cazzola P, Riboni R, Biagi F, Carsetti R (2006). Splenic hypofunction and the spectrum of autoimmune and malignant complications in celiac disease. Clin Gastroenterol Hepatol.

[27] Di Sabatino A, Brunetti L, Maffè GC, Giuffrida P, Corazza GR (2013). Is it worth investigating splenic function in patients with celiac disease?. World J Gastroenterol.

[28] Di Sabatino A, Carnevale Maffè G, Brunetti L, Guerci M, Corazza GR (2013). Splenic hypofunction in patients with an incidental finding of small-sized spleen at abdominal ultrasound. Intern Emerg Med.

[29] Ludvigsson JF, Bai JC, Biagi F, Card TR, Ciacci C, Ciclitira PJ (2014). Diagnosis and management of adult coeliac disease: guidelines from the British Society of Gastroenterology. Gut.

[30] Malandrino N, Capristo E, Farnetti S, Leggio L, Abenavoli L, Addolorato G (2008). Metabolic and nutritional features in adult celiac patients. Dig Dis.

[31] Wierdsma NJ, van Bokhorst-de van der Schueren MAE, Berkenpas M, Mulder CJJ, van Bodegraven AA (2013). Vitamin and mineral deficiencies are highly prevalent in newly diagnosed celiac disease patients. Nutrients.

[32] Simons M, Scott-Sheldon LAJ, Risech-Neyman Y, Moss SF, Ludvigsson JF, Green PHR (2018). Celiac disease and increased risk of pneumococcal infection: a systematic review and meta-analysis. Am J Med.

[33] van Gils T, Nijeboer P, van Wanrooij RL, Bouma G, Mulder CJJ (2015). Mechanisms and management of refractory coeliac disease. Nat Rev Gastroenterol Hepatol.

[34] Al-Toma A, Volta U, Auricchio R, Castillejo G, Sanders DS, Cellier C (2019). European Society for the Study of Coeliac Disease (ESsCD) guideline for coeliac disease and other gluten-related disorders. United Eur Gastroenterol J.

[35] Kennedy NA, Jones GR, Lamb CA, Appleby R, Arnott I, Beattie RM (2020). British Society of Gastroenterology guidance for management of inflammatory bowel disease during the COVID-19 pandemic. Gut.

[36] Donato MF, Invernizzi F, Lampertico P, Rossi G (2020). Health status of liver transplanted patients during the coronavirus outbreak in Italy: a large single center experience from Milan. Clin Gastroenterol Hepatol..

[37] Elli L, Scaramella L, Lombardo V, Scricciolo A, Doneda L, Roncoroni L (2020). Refractory celiac disease and COVID-19 outbreak: findings from a high incidence scenario in Northern Italy. Clin Res Hepatol Gastroenterol.

[38] Morreale F, Agnoli C, Roncoroni L, Sieri S, Lombardo V, Mazzeo T (2018). Are the dietary habits of treated individuals with celiac disease adherent to a Mediterranean diet?. Nutr Metab Cardiovasc Dis.

[39] Sofi F, Macchi C, Abbate R, Gensini GF, Casini A (2013). Mediterranean diet and health status: an updated meta-analysis and a proposal for a literature-based adherence score. Public Health Nutr.

[40] Menotti A, Puddu PE (2015). How the seven countries study contributed to the definition and development of the Mediterranean diet concept: a 50-year journey. Nutr Metab Cardiovasc Dis.

[41] Billingsley HE, Carbone S, Lavie CJ (2018). Dietary fats and chronic noncommunicable diseases. Nutrients.

[42] Ortega RM (2006). Importance of functional foods in the Mediterranean diet. Public Health Nutr..

[43] Gul K, Singh AK, Jabeen R (2016). Nutraceuticals and functional foods: the foods for the future world. Crit Rev Food Sci Nutr.

[44] Wan MLY, Co VA, El-Nezami H (2020). Dietary polyphenol impact on gut health and microbiota. Crit Rev Food Sci Nutr..

[45] Maynard CL, Elson CO, Hatton RD, Weaver CT (2012). Reciprocal interactions of the intestinal microbiota and immune system. Nature.

[46] Bascuñán KA, Araya M, Roncoroni L, Doneda L, Elli L (2020). Dietary gluten as a conditioning factor of the gut microbiota in celiac disease. Adv Nutr.

[47] Sharifi-Rad M, Anil Kumar NV, Zucca P, Varoni EM, Dini L, Panzarini E (2020). Lifestyle, oxidative stress, and antioxidants: back and forth in the pathophysiology of chronic diseases. Front Physiol..

[48] Li P, Yin YL, Li D, Kim WS, Wu G (2007). Amino acids and immune function. Br J Nutr.

[49] Hachimura S, Totsuka M, Hosono A (2018). Immunomodulation by food: Impact on gut immunity and immune cell function. Biosci Biotechnol Biochem.

[50] Carding S, Verbeke K, Vipond DT, Corfe BM, Owen LJ (2015). Dysbiosis of the gut microbiota in disease. Microb Ecol Health Dis..

[51] Chen P, Mao L, Nassis GP, Harmer P, Ainsworth BE, Li F (2020). Coronavirus disease (COVID-19): the need to maintain regular physical activity while taking precautions. J Sport Health Sci.

[52] Hollander JE, Carr BG (2020). Virtually perfect? Telemedicine for Covid-19. N Engl J Med..

[53] Greenhalgh T, Wherton J, Shaw S, Morrison C (2020). Video consultations for covid-19. BMJ.

[54] Siniscalchi M, Zingone F, Savarino EV, D’Odorico A, Ciacci C (2020). COVID-19 pandemic perception in adults with celiac disease: an impulse to implement the use of telemedicine. Dig Liver Dis..

[55] Villani A, Scalvenzi M, Fabbrocini G (2020). Teledermatology: a useful tool to fight COVID-19. J Dermatol Treat..

[56] Cebolla Á, Moreno M, de Coto L, Sousa C (2018). Gluten immunogenic peptides as standard for the evaluation of potential harmful prolamin content in food and human specimen. Nutrients..

[57] Costa S, Astarita L, Ben-Hariz M, Currò G, Dolinsek J, Kansu A (2014). A point-of-care test for facing the burden of undiagnosed celiac disease in the Mediterranean area: a pragmatic design study. BMC Gastroenterol..

[58] Tangermann P, Branchi F, Itzlinger A, Aschenbeck J, Schubert S, Maul J (2019). Low sensitivity of simtomax point of care test in detection of celiac disease in a prospective Multicenter Study. Clin Gastroenterol Hepatol.

[59] Branchi F, Ferretti F, Orlando S, Tontini GE, Penagini R, Vecchi M (2020). Small-bowel capsule endoscopy in patients with celiac disease, axial versus lateral/panoramic view: results from a prospective randomized trial. Dig Endosc.

[60] Elli L, Rimondi A, Scaramella L, Topa M, Vecchi M, Mangioni D (2020). Endoscopy during the Covid-19 outbreak: experience and recommendations from a single center in a high-incidence scenario. Dig Liver Dis.

[61] Tontini GE, Manfredi G, Orlando S, Neumann H, Vecchi M, Buscarini E (2019). Endoscopic ultrasonography and small-bowel endoscopy: present and future. Dig Endosc.

[62] Penny HA, Raju SA, Sanders DS (2020). Progress in the serology-based diagnosis and management of adult celiac disease. Expert Rev Gastroenterol Hepatol.

[63] Husby S, Koletzko S, Korponay-Szabó I, Kurppa K, Mearin ML, Ribes-Koninckx C (2020). European Society Paediatric Gastroenterology, Hepatology and nutrition guidelines for diagnosing coeliac disease 2020. J Pediatr Gastroenterol Nutr.

