# Correction to: How to manage celiac disease and gluten-free diet during the COVID-19 era: proposals from a tertiary referral center in a high-incidence scenario

**DOI:** 10.1186/s12876-021-02092-x

**Published:** 2022-02-28

**Authors:** Luca Elli, Donatella Barisani, Valentina Vaira, Maria Teresa Bardella, Matilde Topa, Maurizio Vecchi, Luisa Doneda, Alice Scricciolo, Vincenza Lombardo, Leda Roncoroni

**Affiliations:** 1 Center for Prevention and Diagnosis of Celiac Disease, Gastroenterology and Endoscopy Unit, Fondazione IRCCS Ca’ Granda Ospedale Maggiore Policlinico, Via F. Sforza 35, 20122 Milan, Italy; 2 Department of Pathophysiology and Transplantation, University of Milano, Milan, Italy; 3 School of Medicine and Surgery, University of Milano-Bicocca, Monza, Italy; 4 Division of Pathology, Fondazione IRCCS Ca’ Granda Ospedale Maggiore Policlinico, Milan, Italy; 5 Department of Biomedical, Surgical and Dental Sciences, University of Milan, Milan, Italy

## Correction to: BMC Gastroenterol (2020) 20:387 10.1186/s12876-020-01524-4

After publication of this article [1], it was reported that the title of reference [51] was incorrect, and should have been “Coronavirus disease (COVID-19): The need to maintain regular physical activity while taking precautions”. Also the doi of this article has been added.

The original article [1] has been updated.
